# Comparative cost analysis of implanting devices in different cardiac resynchronization therapeutic strategies

**DOI:** 10.1002/clc.24107

**Published:** 2023-08-03

**Authors:** Mengna Chen, Jing Shi, Yimin Zhang, Xiaozhen Ge, Xu Zhang, Wenbin Fan, Shuo Wang, Zhiqin Guo, Jian Guan, Yongquan Wu, Junmeng Zhang

**Affiliations:** ^1^ The First Hospital of Tsinghua University Beijing China; ^2^ Beijing Anzhen Hospital Capital Medical University Beijing China

**Keywords:** biventricular pacing, cardiac resynchronization therapy, conduction system pacing, cost analysis, pacemakers

## Abstract

**Background:**

Cardiac resynchronization therapy (CRT) is an established treatment option for heart failure patients. However, the implementation of triple‐chamber pacemakers can be cost‐prohibitive. His‐Purkinje system pacing (HPSP) can also enable cardiac resynchronization, and it can be achieved with relatively inexpensive conventional pacemakers.

**Hypothesis:**

This article aims to comparatively evaluate the cost of implanting devices in different CRT strategies to provide meaningful guidance for clinical decision‐making by electrophysiologists.

**Methods:**

Data was collected on the prices, designed life, and price/designed life of multiple mainstream models of CRT‐P, CRT‐D, dual‐chamber pacemakers, and single‐chamber pacemakers that were sold in the Chinese market in 2022. The prices, designed lives, and price/designed life of different pacemaker models were then compared.

**Results:**

The costs of CRT‐P and CRT‐D (13008.44 ± 2752.30 USD and 22043.36 ± 3676.25 USD) were significantly higher than those of conventional pacemakers (dual‐chamber: 11142.39 ± 4273.85 USD and single‐chamber: 5634.28 ± 2032.80 USD) (*p* < .05). Additionally, the price/designed life of conventional pacemakers (dual‐chamber: 839.63 ± 258.62 US dollar/year and single‐chamber: 435.86 ± 125.44 US dollar/year) was significantly better than that of CRT‐P and CRT‐D (1386.91 ± 266.73 and 2585.53 ± 520.27 US dollar/year, respectively) (*p* < .05).

**Conclusion:**

Conduction system pacing (CSP)‐based CRT is more cost‐effective than BVP‐based CRT. Furthermore, CSP‐based CRT can achieve cardiac resynchronization with conventional pacemakers and may be a good option for HF patients who do not need defibrillation.

## Graphical abstract

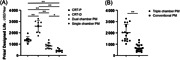

## 1 INTRODUCTION

Cardiac resynchronization therapy (CRT) is considered an effective treatment for patients with congestive heart failure (CHF) and broad QRS duration, LBBB QRS morphology, and left ventricular ejection fraction (LVEF) ≤35%.^1^ CRT based on conventional biventricular pacing (BVP) has been studied in many clinical trials, and it is associated with improved hospitalization and mortality rates among HF patients.^2^ BVP can achieve cardiac electromechanical resynchronization and is the cornerstone of CRT.

Although BVP‐based CRT is widely accepted, the implant rate of CRT in heart failure (HF) patients who are indicated for CRT and successfully treated is very low. Lars and colleagues reported that only 21.4% of HF patients accepted CRT (841 patients accepted CRT among 3935 patients who were indicated for CRT) according to results from the Swedish Heart Failure Registry.^3^ In addition to the technical difficulty of BVP‐based CRT operation, the high cost of triple‐chamber pacemakers is also an important factor that limits their application. Unfortunately, many economically disadvantaged patients have to give up reasonable treatment due to their inability to afford the high cost of CRT. In the end, they have to endure the suffering caused by heart failure, or even death.

Fortunately, the His‐Purkinje system pacing (HPSP) developed recently has been proven to achieve cardiac resynchronization. More importantly, it can achieve ventricular synchronous contraction by using conventional pacemakers (dual‐chamber or single‐chamber pacemakers). HPSP, also called conduction system pacing (CSP), includes His bundle pacing (HBP) and left bundle branch pacing (LBBP). CSP significantly shortens the QRS duration, improves the LVEF, and reduces the incidence of primary endpoints, resulting in improved clinical outcomes.^4^, ^5^ Both HBP and LBBP can successfully achieve CRT.^6^, ^7^, ^8^

**Figure 1:**
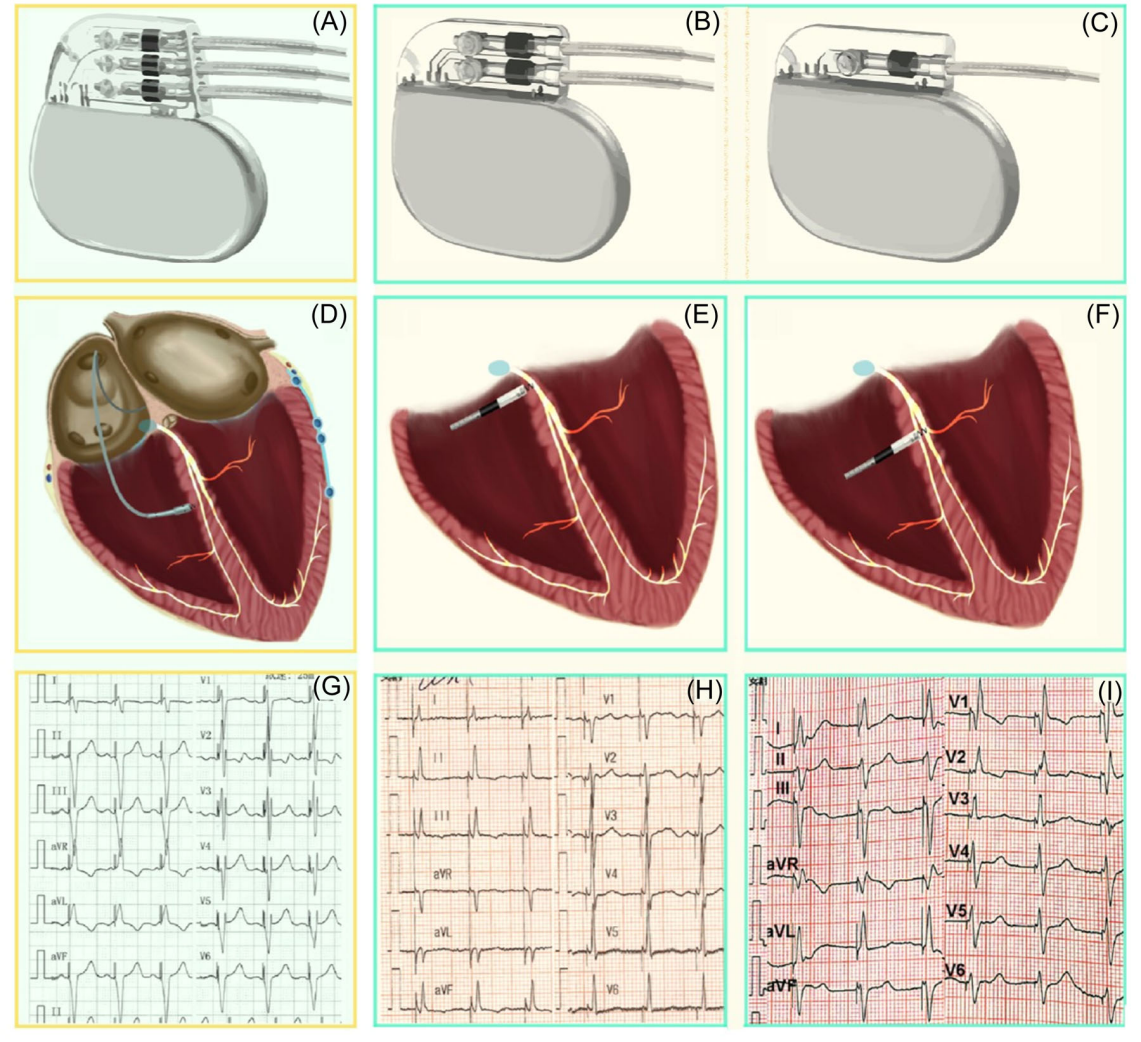
The devices, positions of pace leads in the heart, and ECGs of different CRT strategies. (A) Schematic diagram of a triple‐chamber pacemaker. (B) Schematic diagram of a dual‐chamber pacemaker. (C) Schematic diagram of a single‐chamber pacemaker. (D) Conventional CRT using BVP based on right ventricular pacing and coronary venous pacing. (E) HBP is the real physiological pacing modality that directly activates the conduction bundle. (F) LBBP can directly activate the native left bundle branch region. (G) The 12‐lead ECG after BVP; the paced QRS duration was 134 ± 15 ms.^9^ (H) For the 12‐lead ECG after HBP, the paced QRS duration was 103.8 ± 13 ms.^10^ (I) For the 12‐lead ECG after LBBP, the paced QRS duration was 114.1 ± 10.7 ms.^10^ BVP, biventricular pacing; CRT, cardiac resynchronization therapy; ECG, electrocardiogram; HBP, His bundle pacing; LBBP, left bundle branch pacing.

The devices used in BVP‐based CRT (Figure 1D) are triple‐chamber pacemakers, CRT‐pacemakers (CRT‐P), and CRT‐defibrillators (CRT‐D) (Figure 1A), and these devices are relatively expensive. However, HBP (Figure 1E) and LBBP (Figure 1F) can be used to achieve CRT with relatively inexpensive conventional dual‐chamber (Figure 1B) or single‐chamber pacemakers (Figure 1C).

To date, there has been no study to directly compare the cost analysis of different CRT strategies. Therefore, we collected and analyzed data on the prices, designed lives, and price/designed life of mainstream models of CRT‐P, CRT‐D, dual‐chamber pacemakers, and single‐chamber pacemakers that were sold in the Chinese market. Then, we comparatively studied the cost analysis of different CRT strategies to provide meaningful guidance for decision‐making by electrophysiologists.

## 2 METHODS

We collected data on the prices, designed lives, and price/designed life of multiple mainstream models of CRT‐P, CRT‐D, dual‐chamber pacemakers, and single‐chamber pacemakers from Medtronic, Abbott, Boston Scientific, and Biotronik that were sold in the Chinese market in 2022 (Table 1). The prices of CRT‐P (*n* = 8), CRT‐D (*n* = 9), dual‐chamber pacemakers (*n* = 11), and single‐chamber pacemakers (*n* = 9) were converted from Chinese Yuan to US dollars at an exchange rate of 6.8171 on August 21, 2022. Then, we compared the prices, designed lives, and price/designed life of the different pacemaker models.

**Table 1:** Comparison of different types of pacemakers.

Corporation	Type	Category	Price (US dollar)	Design life (year)	Applicable procedure
Medtronic	C5TR01	CRT‐P	11 735	6~10	BVP/LOT‐CRT/HOT‐CRT
Medtronic	C2TR01	CRT‐P	8215	6~10	BVP/LOT‐CRT/HOT‐CRT
Medtronic	DTBC2QQ	CRT‐D	18 336	6~12	BVP/LOT‐CRT/HOT‐CRT and ICD
Medtronic	DTBA2QQ	CRT‐D	24 204	6~12	BVP/LOT‐CRT/HOT‐CRT and ICD
Medtronic	DTBA2D4	CRT‐D	23 104	6~12	BVP/LOT‐CRT/HOT‐CRT and ICD
Medtronic	A3DR01	Dual‐chamber	8787	8~10	HBP and LBBP
Medtronic	X3DR01	Dual‐chamber	13 187	15	HBP and LBBP
Medtronic	SEDRL1	Dual‐chamber	6454	10~12	HBP and LBBP
Medtronic	EN1SR01	Single‐chamber	6132	8~10	HBP and LBBP
Medtronic	X3SR01	Single‐chamber	8772	15	HBP and LBBP
Medtronic	SESR01	Single‐chamber	3503	8	HBP and LBBP
Abbott	PM3262	CRT‐P	17 603	9	BVP/LOT‐CRT/HOT‐CRT
Abbott	PM3160	CRT‐P	13 202	10	BVP/LOT‐CRT/HOT‐CRT
Abbott	PM3242	CRT‐P	11 295	9	BVP/LOT‐CRT/HOT‐CRT
Abbott	CD3371‐40Q	CRT‐D	23 412	8	BVP/LOT‐CRT/HOT‐CRT and ICD
Abbott	CD3371‐40	CRT‐D	26 111	8	BVP/LOT‐CRT/HOT‐CRT and ICD
Abbott	CD3367‐40QC	CRT‐D	17 603	10	BVP/LOT‐CRT/HOT‐CRT and ICD
Abbott	PM2282	Dual‐chamber	20 537	15	HBP and LBBP
Abbott	PM2182	Dual‐chamber	13 187	15	HBP and LBBP
Abbott	PM2272	Dual‐chamber	16 136	14	HBP and LBBP
Abbott	PM1182	Single‐chamber	8068	18	HBP and LBBP
Abbott	PM1172	Single‐chamber	7334	18	HBP and LBBP
Abbott	PM1124	Single‐chamber	4694	16	HBP and LBBP
Boston Scientific	U128	CRT‐P	14 440	11	BVP/LOT‐CRT/HOT‐CRT
Boston Scientific	G148	CRT‐D	16 480	8	BVP/LOT‐CRT/HOT‐CRT and ICD
Boston Scientific	L131	Dual‐chamber	9182	15	HBP and LBBP
Boston Scientific	S722	Dual‐chamber	8633	15	HBP and LBBP
Boston Scientific	S701	Single‐chamber	3155	10	HBP and LBBP
Biotronik	Edora 8HF‐T QP	CRT‐P	14 522	10	BVP/LOT‐CRT/HOT‐CRT
Biotronik	Edora 8HF‐T	CRT‐P	13 055	10	BVP/LOT‐CRT/HOT‐CRT
Biotronik	Rivacor 7 HF‐T QP	CRT‐D	26 404	9	BVP/LOT‐CRT/HOT‐CRT and ICD
Biotronik	Iforia7 HF‐T	CRT‐D	22 737	7.5	BVP/LOT‐CRT/HOT‐CRT and ICD
Biotronik	Edora 8 DR	Dual‐chamber	11 002	12	HBP and LBBP
Biotronik	Evia DR	Dual‐chamber	7995	12	HBP and LBBP
Biotronik	Estella DR	Dual‐chamber	7467	12	HBP and LBBP
Biotronik	Evia SR	Single‐chamber	4283	12	HBP and LBBP
Biotronik	Estella SR	Single‐chamber	4767	12	HBP and LBBP

Abbreviations: BVP, biventricular pacing; CRT‐D, cardiac resynchronization therapy defibrillator; CRT‐P, cardiac resynchronization therapy pacemaker; HBP, His bundle pacing; HOT‐CRT, His‐optimized CRT; ICD, implantable cardioverter‐defibrillator; LBBP, left bundle branch pacing; LOT‐CRT, left bundle branch‐optimized CRT.

### 2.1 Statistical analysis

SPSS 26.0 was used for statistical analysis in this study. Measurement data are expressed as x ± s. Comparisons among multiple groups of independent samples were conducted in pairs. One‐way ANOVA was used for normally distributed data, and a nonparametric test was used for nonnormally distributed data. Two groups of independent samples were compared in pairs. Data with a normal distribution were analyzed by *t* test, and data with a nonnormal distribution were analyzed by nonparametric test. *p* < .05 was considered statistically significant.

## 3 RESULTS

### 3.1 Price of different pacemakers

**Figure 2:**
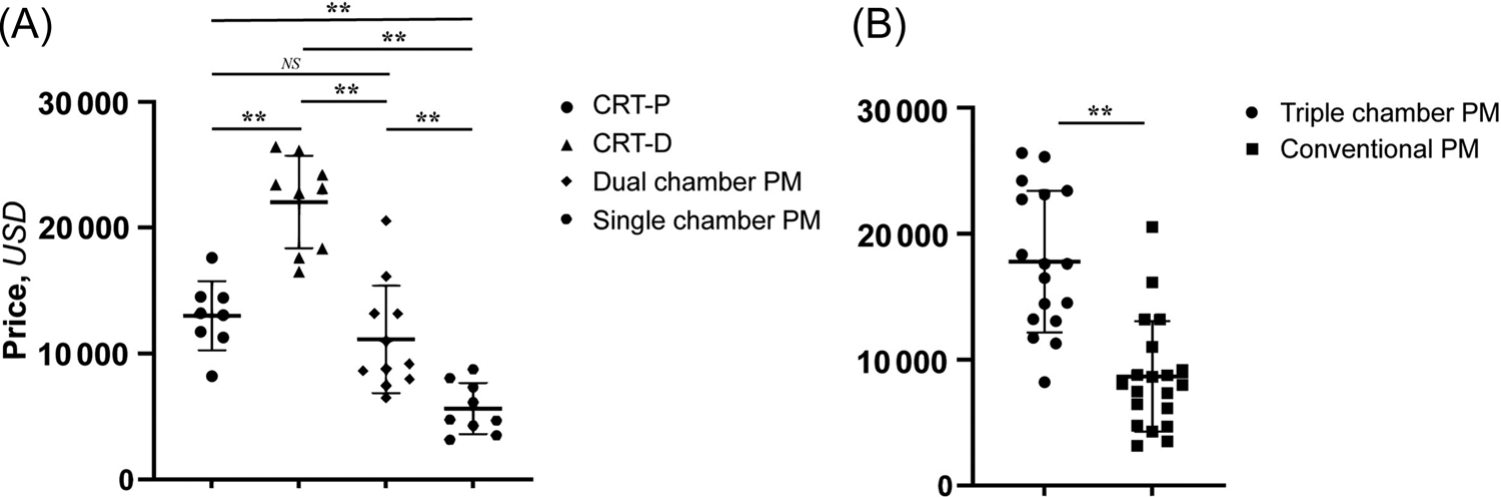
Price comparison of different types of pacemakers. (A) Comparison of the prices of different types of pacemakers (unit: USD). (B) Comparison of the prices of triple‐chamber pacemakers and conventional pacemakers (unit: USD). *<0.05, **<0.01. Triple‐chamber pacemakers include the CRT‐D and CRT‐P; conventional pacemakers include dual‐chamber and single‐chamber pacemakers. CRT‐D, cardiac resynchronization therapy‐ defibrillator; CRT‐P, cardiac resynchronization therapy‐pacemaker; NS, no statistical differences.

The average price of mainstream models of CRT‐P, CRT‐D, dual‐chamber pacemakers, and single‐chamber pacemakers made by Medtronic, Abbott, Boston Scientific, and Biotronik and sold in the Chinese market were 13008.44 ± 2752.30 USD, 22043.36 ± 3676.25 USD, 11142.39 ± 4273.85 USD, and 5634.28 ± 2032.80 USD, respectively. The average price of CRT‐D > CRT‐P > dual‐chamber pacemakers > single‐chamber pacemakers. There were differences among the groups (*p* < .05). (Table 2 and Figure 2A) Triple‐chamber pacemakers were more expensive than conventional pacemakers, and the difference was statistically significant (*p* < .05) (Table 2 and Figure 2B).

**Table 2:** Comparison of prices, designed lives, and price/designed life of different types of pacemakers.

Projects	Triple‐chamber pacemakers		Conventional pacemakers		*p* Value
	CRT‐P	CRT‐D	Dual‐chamber	Single‐chamber	
Price (US dollar)	17791.63 ± 5628.44		8663.74 ± 4388.36		.000
	13008.44 ± 2752.30	22043.36 ± 3676.25	11142.39 ± 4273.85	5634.28 ± 2032.80	.000
Designed life (years)	8.97 ± 0.98		13.15 ± 2.89		.000
	9.38 ± 1.06	8.61 ± 0.78	13.18 ± 2.09	13.11 ± 3.79	.000
Price/designed life	2021.47 ± 739.43		657.93 ± 290.35		.000
	1386.91 ± 266.73	2585.53 ± 520.27	839.63 ± 258.62	435.86 ± 125.44	.000

*Note*: One‐way analysis of variance (ANOVA) was used to compare the differences between the means of multiple independent samples. Two groups of independent samples were compared in pairs. Data with a normal distribution were analyzed by *t* test, and data with a nonnormal distribution were analyzed by nonparametric test. *p* < .05 was considered statistically significant.

Abbreviations: CRT‐D, cardiac resynchronization therapy‐defibrillator; CRT‐P, cardiac resynchronization therapy‐pacemaker.

### 3.2 Designed lives of different pacemakers

**Figure 3:**
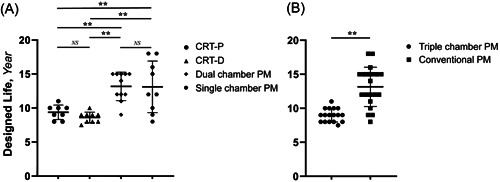
Designed life comparison of different types of pacemakers. (A) Comparison of the designed lives of different types of pacemakers (unit: years). (B) Comparison of the designed lives of triple‐chamber pacemakers and conventional pacemakers (unit: years). *<0.05, **<0.01. Triple‐chamber pacemakers include the CRT‐D and CRT‐P; conventional pacemakers include dual‐chamber and single‐chamber pacemakers. CRT‐D, cardiac resynchronization therapy‐ defibrillator; CRT‐P, cardiac resynchronization therapy‐pacemaker; NS, no statistical differences.

The designed lives of the mainstream models of CRT‐P, CRT‐D, dual‐chamber pacemakers, and single‐chamber pacemakers made by Medtronic, Abbott, Boston Scientific, and Biotronik were 9.38 ± 1.06 years, 8.61 ± 0.78 years, 13.18 ± 2.09 years, and 13.11 ± 3.79 years, respectively. The designed life of dual‐chamber pacemakers > single‐chamber pacemakers > CRT‐P > CRT‐D. There were significant differences among the groups (*p* < .05). (Table 2 and Figure 3A) Comparing the designed life of triple‐chamber pacemakers with that of conventional pacemakers, triple‐chamber pacemakers had shorter lifespans, and the difference was statistically significant (*p* < .05) (Table 2 and Figure 3B).

### 3.3 Price/designed life of different pacemakers

**Figure 4:**
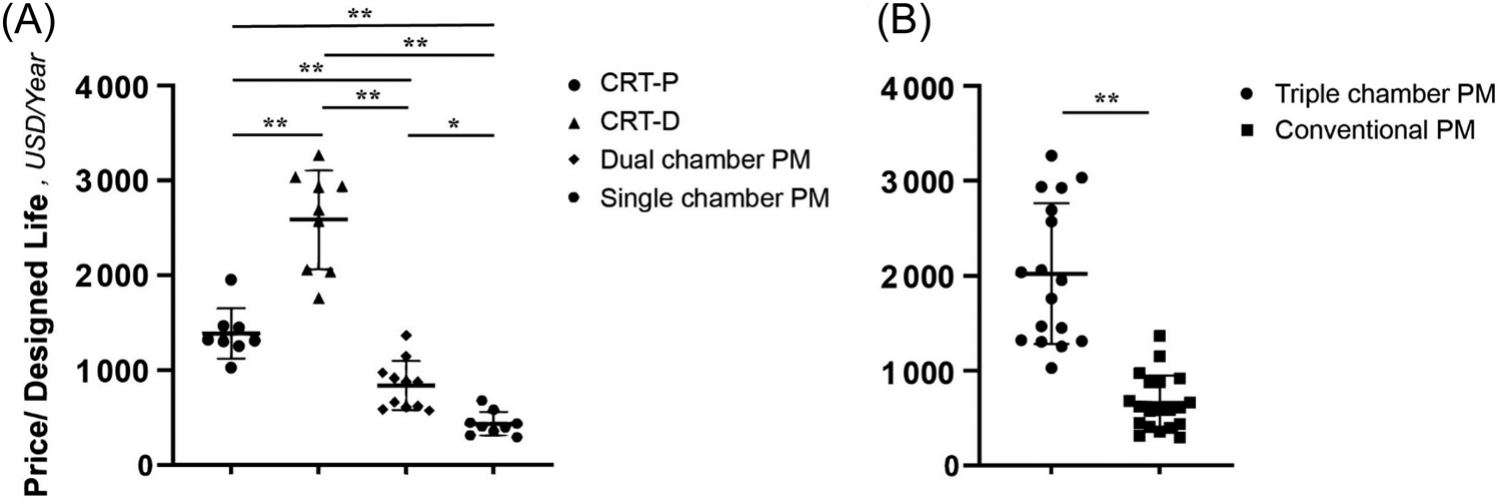
Price/designed life comparison of different types of pacemakers. (A) Comparison of the price/designed life of different types of pacemakers. (B) Comparison of the price/designed life of triple‐chamber pacemakers and conventional pacemakers (unit: USD/years). *<0.05, **<0.01. Triple‐chamber pacemakers include the CRT‐D and CRT‐P; conventional pacemakers include dual‐chamber and single‐chamber pacemakers. CRT‐D, cardiac resynchronization therapy‐ defibrillator; CRT‐P, cardiac resynchronization therapy‐pacemaker.

The price/designed life of the mainstream models of CRT‐P, CRT‐D, dual‐chamber pacemakers, and single‐chamber pacemakers made by Medtronic, Abbott, Boston Scientific, and Biotronik was 1386.91 ± 266.73, 2585.53 ± 520.27, 839.63 ± 258.62, and 435.86 ± 125.44 US dollars/year, respectively. The price/designed life of single‐chamber pacemakers > dual‐chamber pacemakers > CRT‐P > CRT‐D. There were significant differences among the groups (*p* < .05). (Table 2 and Figure 4A) Triple‐chamber pacemakers had lower cost performance than conventional pacemakers, and the difference was statistically significant (*p* < .05) (Table 2 and Figure 4B).

The costs of CRT‐P and CRT‐D were significantly higher than those of conventional pacemakers (*p* < .05), and the price/designed life of conventional pacemakers was significantly better than that of CRT‐P and CRT‐D (*p* < .05).

## 4 DISCUSSION

In this study, we investigated the cost analysis of different CRT strategies. Our findings revealed that the CSP‐based CRT strategy, which only requires a dual‐chamber/single‐chamber pacemaker, has better price advantage in comparison to the BVP‐based CRT strategy, which requires a triple‐chamber pacemaker. These results provide valuable guidance for electrophysiologists in making clinical decisions, especially when patients requiring CRT have financial constraints.

Our analysis indicated that the costs of CRT‐D are higher than that of CRT‐P and dual‐chamber/single‐chamber pacemakers. Single‐chamber pacemakers emerged as the cheapest and most affordable device. This result is consistent with our clinical experience. BVP‐based CRT strategy requires a triple‐chamber pacemaker (CRT‐D/P) to achieve synchronous contraction of the ventricle. In contrast to BVP‐based CRT, either HBP or LBBP can achieve CRT by using conventional pacemakers (dual‐chamber/single‐chamber), which have obvious price advantages. In addition, BVP‐based CRT necessitates the use of three electrodes during implantation, including the expensive left ventricle electrode. In contrast, the 3830 electrode (Medtronic) or Solia S electrode (Biotronik) used in HBP or LBBP is relatively inexpensive. When the number and price difference of upper electrodes are considered, the cost of BVP is higher.

In terms of pacemaker function, CRT‐P abandoned the defibrillation function, while CRT‐D added this function, which is suitable for CRT patients with malignant arrhythmia or the risk of sudden cardiac death, as they have no alternative options. If a single‐chamber pacemaker can achieve CSP‐CRT, it is undoubtedly the most price advantage choice. Unfortunately, this strategy is not feasible for patients requiring atrioventricular synchronized pacing. Therefore, CSP‐CRT with a single‐chamber pacemaker is only suitable for a minority of patients with atrial fibrillation.

The cost of BVP is significantly higher than that of physiological pacemakers, and the designed lives of CRT‐P or CRT‐D (6–10 years) are generally shorter than those of conventional pacemakers (minimum of 10–15 years). The actual cost of conventional BVP is higher when medical costs of later replacements are considered. This is also consistent with our research findings.

Several current clinical CRT strategies have been shown to be effective for cardiac electromechanical resynchronization.^11^ However, there is a significant difference in the prices of different CRT strategies. Our study found that different CRT strategies have varying levels of cost analysis, as represented by price to lifespan ratio. Conventional pacemakers were found to have better price advantage compared to CRT pacemakers.

BVP has been widely performed and well‐studied in clinical settings, and it is still recognized as the cornerstone for achieving CRT. However, BVP has certain disadvantages, such as stimulation of the diaphragm and high pacing threshold, and it can be affected by anatomical limitations and the condition of the target vessel, which limit its success rate.^12^ Notably, 30%–50% of patients do not respond to BVP.^13^, ^14^ HBP is the most physiologically consistent method of ventricular pacing, and has been shown to improve the long‐term prognosis of HF patients.^6^, ^15^, ^16^ It has some limitations, including high pacing threshold and perceptual abnormalities, and is not suitable for patients with block sites below the His bundle. LBBP has the advantages of simple operation, high success rate, stable parameters, and few complications.^17^, ^18^ HPSP may be a promising option for surgical operations and parameters when conventional methods are not feasible.

## 5 CONCLUSION

Compared with BVP‐based CRT, CSP‐based CRT is more cost effective. Additionally, CSP‐based CRT can achieve cardiac resynchronization with a conventional pacemaker and may be a good option for HF patients who do not need defibrillation, especially for patients who have experienced BVP‐based CRT failure or are unable to afford the price of BVP.

## 6 LIMITATIONS

Only some, not all, pacemaker models were included in this study, and the results may be somewhat biased. In addition, pacemaker prices are influenced by local health care policies.

## CONFLICT OF INTEREST STATEMENT

The authors declare no conflict of interest.

## Data Availability

The data that support the findings of this study are available from the corresponding author upon reasonable request. The data sets used or analyzed during the current study are available from the corresponding author on reasonable request.
